# Genome-Wide Association Study of Conformation Traits in Brazilian Holstein Cattle

**DOI:** 10.3390/ani14172472

**Published:** 2024-08-25

**Authors:** Emanueli F. P. Silva, Rita C. Gaia, Henrique A. Mulim, Luís Fernando Batista Pinto, Laiza H. S. Iung, Luiz F. Brito, Victor B. Pedrosa

**Affiliations:** 1 Department of Animal Sciences, State University of Ponta Grossa, Ponta Grossa 84010-330, PR, Brazil; emanuzootec@gmail.com (E.F.P.S.); rita@apcbrh.com.br (R.C.G.); 2 Department of Animal Sciences, Purdue University, West Lafayette, IN 47907, USA; hmulim@purdue.edu (H.A.M.); britol@purdue.edu (L.F.B.); 3 Department of Animal Sciences, Federal University of Bahia, Salvador 40026-010, BA, Brazil; luisfbp@gmail.com; 4 Neogen Corporation, Pindamonhangaba 12412-800, SP, Brazil; liung@neogen.com; 5 Neogen Corporation, Biotechnology Research, Lincoln, NE 68504, USA

**Keywords:** conformation traits, dairy cows, dairy strength, environment, feet and legs, genes, longevity, mammary system, rump, ssGBLUP

## Abstract

**Simple Summary:**

Conformation traits have been a key breeding goal in dairy cattle for many years due to their relationship with animal productivity and other relevant characteristics. Therefore, the primary objectives of this study were to conduct genome-wide association studies (GWASs) for conformation traits and identify candidate genes and metabolic pathways associated with these traits in Brazilian Holstein cattle. Phenotypic, pedigree, and genomic (100 K SNP chip) data from 2339 Holstein animals raised across multiple Brazilian states were used for this study. Thirty-six significant SNPs were identified for five composite traits. These genomic markers overlap with candidate genes influencing biological processes such as myogenesis, adipogenesis, and angiogenesis. These findings contribute to unraveling the genetic background of conformation traits in Brazilian Holstein cattle.

**Abstract:**

The linear conformation of animals exerts an influence on health, reproduction, production, and welfare, in addition to longevity, which directly affects the profitability of milk-producing farms. The objectives of this study were (1) to perform genome-wide association studies (GWASs) of conformation traits, namely the Rump, Feet and Legs, Mammary System, Dairy Strength, and Final Classification traits, and (2) to identify genes and related pathways involved in physiological processes associated with conformation traits in Brazilian Holstein cattle. Phenotypic and genotypic data from 2339 Holstein animals distributed across the states of Rio Grande do Sul, Paraná, São Paulo, and Minas Gerais were used. The genotypic data were obtained with a 100 K SNP marker panel. The single-step genome-wide association study (ssGWAS) method was employed in the analyses. Genes close to a significant SNP were identified in an interval of 100 kb up- and downstream using the Ensembl database available in the BioMart tool. The DAVID database was used to identify the main metabolic pathways and the STRING program was employed to create the gene regulatory network. In total, 36 significant SNPs were found on 15 chromosomes; 27 of these SNPs were linked to genes that may influence the traits studied. Fourteen genes most closely related to the studied traits were identified, as well as four genes that showed interactions in important metabolic pathways such as myogenesis, adipogenesis, and angiogenesis. Among the total genes, four were associated with myogenesis (*TMOD2*, *TMOD3*, *CCND2*, and *CTBP2*), three with angiogenesis (*FGF23*, *FGF1*, and *SCG3*), and four with adipogenesis and body size and development (*C5H12orf4*, *CCND2*, *EMILIN1*, and *FGF6*). These results contribute to a better understanding of the biological mechanisms underlying phenotypic variability in conformation traits in Brazilian Holstein cattle.

## 1. Introduction

Genetic selection has played a major role in increasing the sustainability of the dairy farming sector around the world. Production efficiency has improved over the last decade, with an expressive increase in milk production over the past few decades [1]. However, concern about exclusively selecting animals with a genetic profile aimed at increasing milk production has led to unbalanced genetic progress between traits of economic interest [2]. Among these traits, linear conformation traits show a positive genetic correlation with milk production traits in dairy cattle [2,3]. Furthermore, they influence important aspects of dairy farming, including animal health, udder health, and reproductive traits such as calving ease. Thus, conformation traits are associated with both productive and functional traits [4].

Conformation traits have a correlation (0.15 to 0.40) with cows’ functional longevity [5]. Significant single-nucleotide polymorphisms (SNPs) and candidate genes linked to udder quality, body conformation, and fitness in different Holstein cattle populations have been found by genome-wide association studies (GWASs) for conformation traits. SNPs related to udder structure features, such as anterior udder attachment, udder depth, and posterior udder attachment height, have been reported in studies evaluating Chinese Holstein cattle [6,7]. Furthermore, SNPs associated with body depth, fore udder attachment, and overall score have been identified in Czech Holsteins [8]. The adaptation of animals to different environments, diseases, and parasites allows the identification of genes involved in the adaptive process within the species’ genome [9]. Therefore, the evaluation of breeds in different environments is important. Some studies using the GWAS approach in Brazilian Holstein and Girolando dairy cattle breeds [10,11,12] have reported important genomic regions associated with milk production traits, but such results have not yet been reported for conformation traits.

In Brazilian Holstein cattle, there are some important scores used in morphological evaluations. The “Final Classification” (FC) score assess the animal’s general body structure [13]. The “Dairy Strength” (DS) trait assesses the stature, chest width, body depth, angularity, body condition, and topline leveling of the animal, enabling an evaluation of its body balance and production capacity [14], as well-conformed cows likely have greater productive potential and functional longevity. The “Rump” trait is based on a group of variables including rump width, rump angle, loin strength, udder width, and udder attachment [14]. “Feet and Legs” (FL) is assessed based on bone quality, side- and rear-view observations of the legs, heel depth, and hoof angle, thus assessing locomotion characteristics. The “Mammary System” (MS) score includes udder height, udder width, teat placement observed from the rear view, central ligament, udder texture, udder depth, udder attachment, front teat placement, and teat length. MS is associated with cow longevity, health, and well-being, and, therefore, is directly linked to economic returns [15].

In artificial selection, the weight attributed to each trait in breeding schemes reflects the herd’s requirements and breeding objectives, as well as consumer preferences and social needs such as aspects involving the environment [5]. The incorporation of genomic data into genomic prediction analyses has resulted in greater genetic progress compared to pedigree-based selection [16]. An important genomic approach is the genome-wide association study (GWAS) method. One of the strategies of this approach is the investigation of genomic variants that are associated with traits of economic interest [7,17] through the exploration of genomic architecture [16] using SNPs as markers. This technique shows good performance in the assessment of the genetic bases of complex traits, i.e., traits influenced by various genes, such as conformation traits [18].

Gene ontology (GO) enrichment analyses are used to determine the roles of genes that overlap or are in linkage disequilibrium with SNPs associated with enriched pathways that may be related to the traits of interest [19]. Several candidate genes, including *MTUS1*, *PRKN*, *MGST1*, *MGST2*, *STXBP6*, and many more, have been linked to udder characteristics [6]. Moreover, conformational features of the legs and feet of Holstein cattle have been found to be associated with genes such as *ADIPOR2*, *INPP4A*, *DNMT3A*, and *ALDH1A2* [20]. The genetic architecture of conformation traits in Holstein cattle is better understood thanks to these findings, which also enhances animal longevity, productivity, and health in the dairy sector. The objectives of the present study were (1) to perform GWASs of conformation traits, namely the Rump, Feet and Legs, Mammary System, Dairy Strength, and Final Classification traits, and (2) to identify candidate genes involved in biological processes associated with conformation traits in Brazilian Holstein cattle.

## 2. Materials and Methods

### 2.1. Phenotypes and Genotypes

We used data on conformation traits measured in 2339 genotyped Holstein cows from herds in the Brazilian states of Minas Gerais, Paraná, Rio Grande do Sul, and São Paulo. Phenotypic data were collected from primiparous cows in 2021 and 2022. Genotypic data were obtained with the GGP Bovine 100K SNP chip (Neogen Corporation, Lincoln, NE, USA). The genetic material was extracted from hair sample follicles using the phenol–chloroform extraction protocol [21]. A total of 3936 animals were included in the pedigree file after data editing.

The composite traits investigated [22] were as follows:

- FC, denoting the final score of all conformation traits;
- DS, comprising the animal’s stature, topline leveling, chest width, body depth, angularity, body condition score, bone quality, udder texture, and lumbar strength;
- Rump, considering the croup angle, croup width, loin strength, and bone quality;
- FL, considering the legs from a side view, hoof angle, heel depth, and the legs from the back view;
- MS, comprising the udder depth, udder texture, median ligament, anterior insertion of the udder, anterior teat placement, udder height, udder width, udder placement, and teat length.

### 2.2. Quality Control

During the genomic quality control process, we removed SNPs with a call rate < 0.90, SNPs located on non-autosomal chromosomes, SNPs with an unknown or duplicated position, SNPs with a minor allele frequency (MAF) < 0.05, and SNPs with high linkage disequilibrium with other SNPs (r^2^ > 0.995). After quality control, 82,897 markers remained for subsequent GWASs.

Phenotypic records that exceeded three standard deviations from the mean within the contemporary group (herd, year, and season of birth) were considered to be outliers and removed from further analyses.

### 2.3. Single-Step GWASs

The single-step GWAS (ssGWAS) method was employed for genomic association analyses using the BLUPF90 family of programs [23]. The RENUMF90 module was used to renumber the phenotypic data and genomic markers. The PREGSF90 module [24] was used for performing genotypic quality control and structuring the relationship matrix, while BLUPF90 [23] was used for the processing of mixed-model equations. Finally, the postGSF90 module was used to back-solve genomic estimated breeding values and to present solutions for the effects of SNPs on each trait.

The traits were analyzed using the following animal model:

y = Xb + Za + e,

where y is the vector of phenotypic observations recorded in primiparous cows; X is the incidence matrix that relates the phenotypes to the fixed effects; b is the vector of fixed effects related to the contemporary group (farm, year, and season of birth) and considering the animal age in months as a linear and quadratic covariate; Z is the incidence matrix that relates the additive genetic effect to the phenotypic records; a is a vector of additive genetic effects; and e is the vector of residual effects.

The variances of a and e are represented as follows:

$$\mathrm{Var}\;[a\;e]=\left[\begin{array}{cc} {H\sigma^{2}}_{a} & 0\\ 0 & {I\sigma^{2}}_{a} \end{array}\right],$$

where σ^2^_a_ is the direct additive genetic variances for each trait; σ^2^_e_ is the residual variance; H is the matrix that combines pedigree and genomic information [25]; and I is an identity matrix. The inverse of the matrix H is represented by the following equation:

$$H^{-1}=A^{-1}+\left[\begin{array}{cc} 0 & 0\\ 0 & G^{-1}-A_{\;22}^{-1} \end{array}\right],$$

where A is the relationship matrix based on the pedigree of all animals; A_22_ is the relationship matrix based on the pedigree of the genotyped animals; and G is the genomic relationship matrix [26].

The following algorithm was used to solve the SNP effects based on the ssGWAS method [27]:

a = Zu,

where a is the vector of breeding values for genotyped individuals calculated using BLUPF90+; Z is a matrix relating individuals to phenotypes; and u is a vector of the effects of SNP markers.

The SNP effects were estimated using the following equation:

û = IZ′(ZIZ′)^−1^â,

where û is a vector of the effects of SNP markers; I is an identity matrix; Z is a matrix that relates the individuals to the phenotypes. Each SNP was assumed to have an equal variance of allele substitution effects and the SNP effects were assumed to follow the infinitesimal model.

The approximate *p*-values of each SNP tested were obtained with the POSTGSF90 program implemented in the BLUPF90 package [28], using the following equation [29]:

$$p_{i}=2\;(1-\Phi \;(\left|\frac{\alpha_{i}}{SD(\alpha_{i})}\right|)),$$

where *α_i_* is the SNP effect estimate; *SD* is the standard deviation; and Φ is the standard normal cumulative distribution function. The *p*-values were generated by back-solving the SNP effects from the estimated breeding values. This approach was possible because fitting the animal as a random effect to generate estimated breeding values is equivalent to fitting all SNPs as random effects and solving those effects directly [30].

### 2.4. Identification of Genes and Functional Analyses

The genomic regions harboring significant SNPs were explored in order to identify genes associated with the studied traits, considering a *p*-value significance threshold of <5 × 10^−8^ [31]. The list of overlapping (or closely related) genes was assembled considering 100 kb up- and downstream of the significant SNP position in the genome, and the previously reported QTLs overlapping with the identified genomic regions were retrieved using the GALLO R package [32]. The Ensembl Genes database and the bovine reference genome ARS-UCD1.2 [33] were used for these functional genomic analyses.

To improve the understanding of the biological processes shared by these annotated genes, enrichment analyses were performed using the functional annotation tools of the DAVID database [34,35]. Additionally, gene networks were generated using the STRING tool [36]. The criteria used to select the genes for analysis were based on the function and products of the gene as described in the literature, and their relationship to the traits under study.

## 3. Results

### 3.1. Descriptive Statistics

The descriptive statistics for the Final Classification (FC), Dairy Strength (DS), Rump, Feet and Legs (FL), and Mammary System (MS) traits of Brazilian Holstein cows are presented in Table 1.

### 3.2. Single-Step GWASs

**Figure 1:**
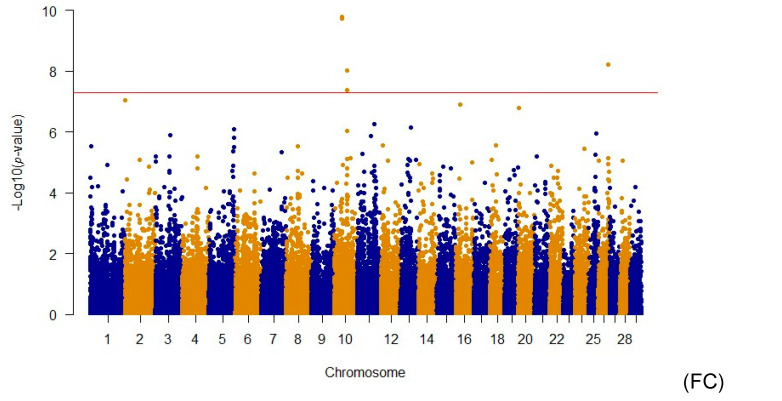

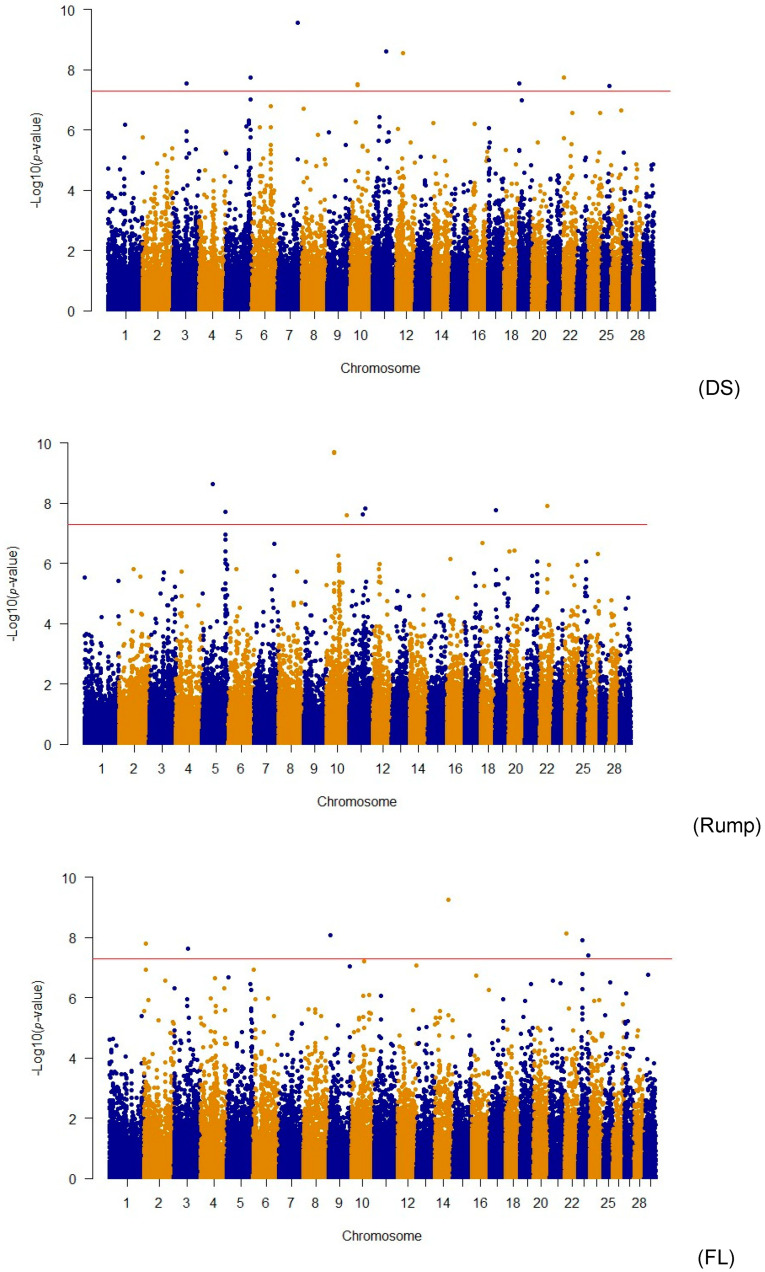

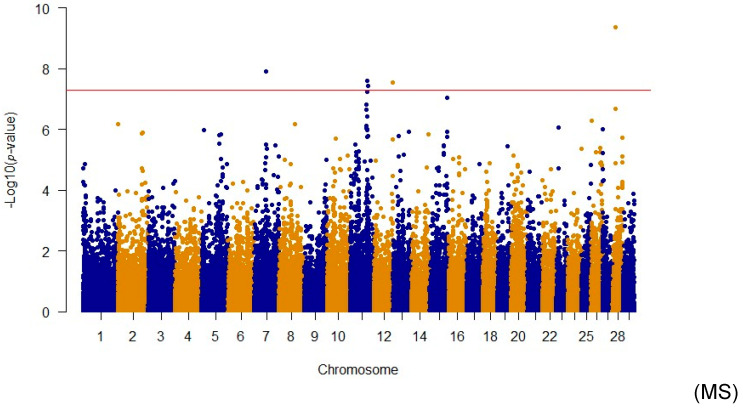
Manhattan plots displaying the association of genomic markers with Final Classification (FC), Dairy Strength (DS), Rump, Feet and Legs (FL), and Mammary System (MS) traits in Brazilian Holstein cattle. The red line indicates the significant threshold for the association with the trait.

GWASs of the conformation traits revealed 36 significant SNPs, including 5 for Final Classification, 10 for Dairy Strength, 9 for Rump, 7 for Feet and Legs, and 5 for Mammary System, as shown in Figure 1. The significant SNPs for the five traits were identified on the following chromosomes: FC: BTA10 and BTA26; DS: BTA3, BTA5, BTA7, BTA10, BTA11, BTA12, BTA19, BTA22, and BTA25; Rump: BTA5, BTA10, BTA11, BTA19, and BTA22; FL: BTA2, BTA3, BTA9, BTA14, BTA22, and BTA23; and MS: BTA7, BTA11, BTA12, and BTA28. Twenty-seven of the identified SNPs were linked to known genes and had QTLs related to conformation traits. Table 2 presents the significant SNPs identified for each trait and the genes associated with them.

### 3.3. Functional Analyses

The GO terms are shown in Table 3. The *TMOD2* and *TMOD3* genes, which play a role in the formation of striated skeletal muscle [37], were associated with the FC. Sixteen genes (*EMILIN1*, *FGF23*, *FGF6*, *TCF23*, *RAD51AP1*, *ACTR2*, *CCND2*, *MAPRE3*, *HELB*, *FRMD4B*, *TIGAR*, *C5H12ofr4*, *KHK*, *ARL6IP5*, *LMOD3*, and *TMAM214*) associated with the formation of the Rump component were enriched for the regulation of cellular processes, the regulation of gene expression, signaling pathways, cell differentiation, organ morphogenesis, and other cellular functions.

Seven of these sixteen genes (*C5H12orf4*, *CCND2*, *EMILIN1*, *FGF23*, *FGF6*, *HELB*, and *TIGAR*) were found to be closely related to the Rump component. For FL, eight genes with functions in the membrane and cytosol were found to be enriched: *ABCC10*, *ADGRL2*, *OSBPL10*, *TJAP1*, *POLH*, *HIVEP1*, *XPO5*, and *ZNF318*. Three of these genes (*POLH*, *HIVP1*, and *ADGRL2*) were associated with FL. Two genes were identified for MS, including *COL4A2* and *FGF1*, which have known functions in the extracellular matrix. No enriched genes associated with GO terms were found for DS.

### 3.4. Gene Interaction Network

**Figure 2:**
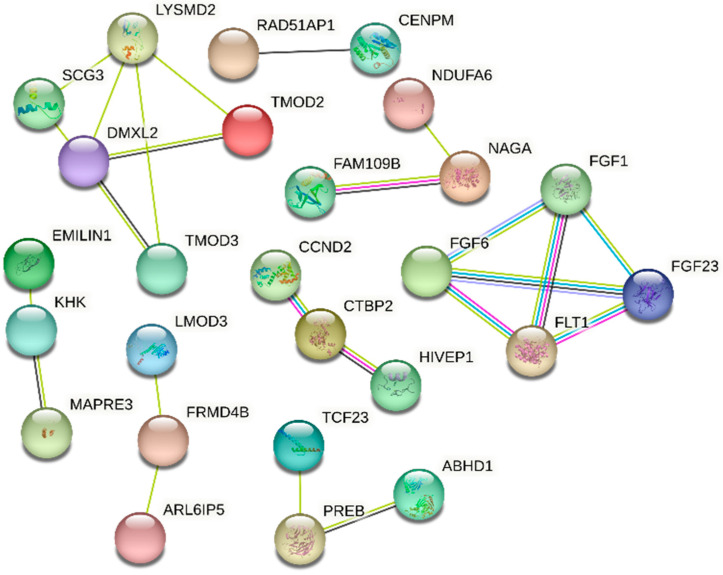
Gene interaction network for the genes associated with conformation traits in Brazilian Holstein cattle.

Figure 2 illustrates the gene interaction network. Several genes with different interaction intensities were identified for the conformation traits. As shown in Figure 2, there was an interaction between the genes for Rump (*FGF6* and *FGF23*) and MS (*FGF1*), as well as with the *FLT1* gene involved in angiogenesis. This is important since the heart is one of the main components of the cow’s milk production system due to its function as a blood pump [38].

The *TMOD2* and *TMOD3* genes, related to Final Classification, interact with the *SCG3* gene (involved in angiogenesis). The genes most closely related to Rump (*CCND2*) and FL (*HIVP1*) show interactions with each other and with the *CTBP2* gene, which is a gene involved in the process of myogenesis. In addition to these interactions, the *EMILIN* and *KHK* genes are associated with Rump and seem to interact among themselves.

## 4. Discussion

### 4.1. Final Classification

The *TMOD2* gene (i.e., tropomodulin 2) is located on BTA10 in cattle. This protein is part of the tropomodulin family, which consists of four isoforms: *TMOD1*, *TMOD2*, *TMOD3*, and *TMOD4* [39]. The *TMOD2* isoform is expressed in neural structures [40,41,42,43] and encodes a neural tropomodulin that regulates the elongation and depolymerization of actin by interacting with tropomyosin [44]. This protein has been suggested to be associated with the formation of new synaptic structures and the extension of neurites (axon or dendrite) [45]. This process is crucial as the formation or extension of axons directly influences their length, connectivity, and overall functionality [46,47].

The *TMOD3* gene (i.e., tropomodulin 3), also located on BTA10, is an isoform of the tropomodulin family [39] and is mainly expressed in skeletal muscle tissue. *TMOD3* encodes a protein that binds to the pointed ends of thin actin filaments [48], covering and linking these filaments [49]. *TMOD3* controls actin filament polymerization [50] and the interaction of actin with tropomyosin [51]. These functions are important for the maintenance of the structure and function of muscle fibers. A recent study has demonstrated associations of this protein with other traits, such as the adaptive response of mice exposed to different atmospheric pressures at different altitudes [49]. Another study associated the *TMOD3* gene with growth traits in dromedaries as a result of its involvement in erythrocyte development, pointed-end actin filament capping, and tropomyosin-binding pathways [52].

The participation of the *TMOD2* and *TMOD3* genes in the process of myogenesis suggests their relationship with muscle development, which can indirectly influence the conformation traits of dairy cattle.

The *CTBP2* gene (i.e., C-terminal-binding protein 2), located on BTA26, encodes C-terminal-binding protein 2, a protein involved in various cellular processes such as transcriptional regulation and DNA damage response. The main function exerted by members of the CTBP family is that of a transcriptional corepressor, interacting with transcription factors and affecting DNA transcription. Additionally, this gene acts in organelles such as the Golgi complex and is involved in the differentiation of brown adipose tissue [53] and cell apoptosis [54]. The *CTBP2* gene is also involved in the transcriptional regulation of Notch mediators and other signaling pathways [55], thus participating in the control of various biological processes [56]. Given its involvement in cellular processes, the *CTBP2* gene affects developmental processes such as myogenesis [57]. Furthermore, some studies suggest that it exerts an influence on the immune [58,59] and reproductive [60,61,62] systems. *CTBP2* has also been reported as a candidate gene for milk protein percentage [63] and angularity in Holstein cows [64].

The *ABHD1* gene (i.e., abhydrolase domain-containing 1) is located on BTA11. This gene has been found close to an SNP associated with growth traits in Charolais beef cattle [65]. In Criollo dairy cows, this gene was identified in a run of homozygosity (ROH) [66].

The mRNAs of this gene have been detected in diverse cell types, such as smooth-muscle cells, fibroblasts, some types of blood cells, endothelial cells, and epithelial cells, [67] as well as cells of the liver, an organ with the activity of the *ABHD1* gene [68]. The liver interferes with body growth and development since it assists and stimulates the growth of cells and tissues such as bones, muscles, and other organs. The proteins/enzymes encoded by this gene are involved in the metabolic processes of fatty-acid degradation in the liver [69,70]. Furthermore, one study on mice revealed that alpha–beta hydrolase-containing domain 1 plays a role in the regulation of plasma levels of lysophosphatidylcholines [71], an important phospholipid component of cell membranes.

### 4.2. Rump

The function of the *C5H12orf4* gene (i.e., chromosome 5 C12orf4), located on BTA5, has not yet been fully elucidated, but it is known to play role in cell differentiation and growth. Studies on cattle have found *C5H12orf4* to be a candidate gene for body size [72] and body weight [73] in different breeds. This fact points to a possible relationship of this gene with the body structure of cattle, indicating a direct relationship with linear conformation in dairy cattle.

The *CCND2* gene (i.e., cyclin D2), located on BTA5, encodes cyclin D2, a regulatory protein that is part of the cyclin D2/cyclin-dependent kinase 4 (*CDK4*) complex. This complex is involved in the transition from the G1 phase to the S phase [74,75,76]. Studies indicate participation of this gene in reproduction and in the regulation of reproductive hormones in different species [77,78,79,80,81,82,83,84]. The *CCND2* gene has also been associated with the proliferation of pre-adipocytes [85,86] and muscle cells [87]. In cattle, this gene has been identified as a possible influencer of stature and body size [72,88,89] and body conformation [90], as well as of daily weight gain in Holstein cattle [73]. As seen in studies evaluating this gene, *CCND2* might be associated with the morphological traits of animals since it is involved in myogenesis and adipogenesis, influencing the composition of body structure.

The *EMILIN1* gene (i.e., elastin microfibril interfacer 1), located on BTA11, encodes an extracellular-matrix glycoprotein. *EMILIN1* is involved in elastogenesis in different tissues, in addition to its interaction with transforming growth factor beta (*TGFβ*) [91], a growth factor that plays a role in embryonic development, cell differentiation, hormone secretion, and immune function. This protein is part of the microfibrillar structure of elastic fibers [92], contributing to the elasticity of skin and tissues [93]. Additionally, the *EMILIN1* gene has been associated with growth traits in Charolais cattle [65] and with placentation and trophoblast migration and invasion [66,94,95]. A higher expression of this gene is observed in tissues that require greater elasticity, such as the skin and organs like the uterus and mammary glands. This gene can affect the conformation of animals since it has been associated with the growth of cattle. The *EMILIN1* gene can also be associated with the maintenance of the Mammary System, which affects dairy cattle.

The *FGF23* gene (i.e., fibroblast growth factor 23), located on BTA5, is a member of the fibroblast growth factor (*FGF*) family. Studies have linked this hormone to different biological processes, such as angiogenesis, morphogenesis, tissue regulation, and oncogenesis [96]; to the cardiovascular system [97]; and to the formation of osteoclasts [98]. Produced mainly in bones, *FGF23* is involved in phosphate homeostasis (PO_4_^3−^) and vitamin D metabolism [99,100,101,102]. PO_4_^3−^ actively participates in various biological processes such as skeletal development, bone mineralization, membrane composition, nucleotide structuring, the maintenance of plasma pH, and cell signaling.

The *FGF6* gene (i.e., fibroblast growth factor 6), located on BTA5, is also a member of the FGF family that is involved in different cellular processes. Studies on different cattle breeds have demonstrated the influence of this gene on body size [72] and body conformation [90], as well as suggesting that this gene has a possible effect on muscle mass [103]. Furthermore, it has been suggested that the levels of expression of this gene in adipose tissue are increased in colder environments for better thermogenesis [104]. This gene is involved in biological processes such as angiogenesis, morphogenesis, tissue regulation, and oncogenesis [96], as well as in embryonic development [105]. Studies on fish have reported the influence of *FGF6* on muscle tissue hyperplasia [106]. Additionally, a very important role of this protein in myogenesis has been observed in both adult and newborn rats [107]. *FGF6* is also involved in muscle regeneration [108,109].

The *HELB* gene (i.e., DNA helicase B) is located on BTA5 and is expressed during cell replication [110] since it is involved in DNA repair and duplication. As a helicase, it unwinds the double-helix DNA and is therefore mainly found in the nucleus during the G1 phase of the cell cycle [111]. Furthermore, in cattle, this gene plays a role in the adaptation of animals to regions with a tropical climate [112] and to environments characterized by constant high temperatures and high levels of UV intensity [113]. Several SNPs in this gene have been associated with yearling weight in Brahman cattle [9].

The *TIGAR* gene (i.e., TP53-induced glycolysis-regulator phosphatase) is located on BTA5 and exerts important functions at the cellular level, being involved in glucose metabolism [114,115,116], the regulation of the cell cycle [117], and cellular responses to metabolic stress [118]. This gene is expressed in almost all tissues of the body, with higher levels of expression in muscles, the brain, and the heart [119], and is mainly regulated by the stress-induced transcription factor p53, which is responsible for controlling cellular mechanisms such as the cell cycle and apoptosis [120]. The *TIGAR* gene encodes a glucose-regulating enzyme that contributes to the formation of nicotinamide adenine dinucleotide phosphate (NADPH) [121]. Furthermore, studies on cattle have associated this gene with body size, body weight, and body structure [122].

The *SCG3* gene (i.e., secretogranin III) is located on BTA10 and encodes secretogranin III, a protein that is mainly expressed in the neuroendocrine system and that plays a role in the storage and transport of neurotransmitters [123]. A recent study has indicated involvement of this gene in angiogenesis [124]. *SCG3* has also been associated with obesity in humans [125] and puberty in cattle [126,127,128].

### 4.3. Feet and Legs

The *POLH* gene (i.e., DNA polymerase eta), located on BTA23, encodes DNA polymerase eta, an enzyme involved in the cell cycle, DNA replication and repair, and cell morphology [129]. Mutations in this gene affect the accuracy of DNA replication, resulting in additional mutations [130,131]. Previous studies in the literature do not establish a direct linkage between the Feet and Legs trait and the genomic regions identified here. However, polymerases play a crucial role in repairing DNA lesions and preventing a wide range of DNA damage, which is vital for embryonic viability [132]. During mitotic division, polymerases, in conjunction with other proteins, ensure the successful completion of DNA replication, which is vital for several biological organizations as well as for body structure [133].

The *HIVEP1* gene (i.e., human immunodeficiency virus type I enhancer-binding protein 1) is located on BTA23. The protein encoded by this gene participates in the regulation of genes involved in the inflammatory response [134,135,136]. 

The *ADGRL2* gene (i.e., adhesion G-protein-coupled receptor L2), located on BTA3, has been associated with weight and growth in pigs [137], as well as with the development of intra- and extracellular regions [138]. In humans, this gene has also been associated with the immune system [139].

### 4.4. Mammary System

The *COL4A2* gene (i.e., collagen type IV alpha 2), located on BTA12, encodes the proα2 (IV) protein, a precursor molecule of type IV collagen responsible for providing structural support to tissues and organs such as the endomysium and perimysium [140]. This type of collagen is the main component of basement membranes [141]. Together with other membrane components, this collagen is responsible for incorporation, regulation, and membrane adhesion [142]. Mutations in this gene can cause diseases in different tissues, including muscle tissue [143].

A study on sheep found an increase in the expression of the *COL4A2* gene at the end of lactation, which was associated with basal lamina turnover during involution of the mammary glands [144]. In milk-producing Holstein cows, this gene was found to play an important role in maintaining the morphology and function of the mammary glands [145]. A study evaluating different types of diet in Holstein cows found a difference in gene expression depending on the type of feed ingested, which influenced mammary growth and development [146]. Furthermore, recent studies indicate an influence of the *COL4A2* gene on fat metabolism [147,148]. According to the traits already related to these genes, their functions are associated with udder constitution/structure in dairy cattle.

The *FGF1* gene (i.e., fibroblast growth factor 1), located on BTA7, is a member of the FGF family. The fibroblast growth factor 1 protein plays a role in the regulation of cell proliferation [149], cell division, cell migration, neurogenesis [150,151], and angiogenesis [152]. The *FGF1* gene is involved in reproduction [153,154,155,156,157] in bovines. Furthermore, its protein is important for tissue repair [158] considering that the FGF group of proteins participates in the formation of a type of connective tissue.

The *FLT1* gene (i.e., vascular endothelial growth factor receptor 1), located on BTA12 and a member of the kinase family, is involved in angiogenesis [159,160,161,162]. Furthermore, recent studies on Holstein cattle have associated this gene with other traits such as udder depth, central ligament, Dairy Strength, and stillbirth risk [163], as well as with the innate immune response [164]. These findings lead us to consider this gene as a candidate for the formation of the traits studied, especially those related to conformation of the Mammary System.

## 5. Conclusions

We identified 36 significant SNPs and 18 candidate genes associated with 23 metabolic pathways for the five traits evaluated. Among the highlighted genes, the *FGF23* gene has been associated with osteogenesis and angiogenesis. The *CCDN2*, *TMOD2*, *TMOD3*, and *CTBP2* genes have been associated with myogenesis. Furthermore, the *C5H12orf4*, *FGF6*, *EMILIN1*, and *ABHD1* genes have been associated with body size, growth, and weight gain in cattle. The *SCG3* and *FLT1* genes have been associated with angiogenesis. The *HELB* and *TIGAR* genes are involved in the adaptation of animals to different climates and altitudes. In conclusion, we identified various genomic regions associated with five conformation traits in Brazilian Holstein cattle. These findings are important for understanding the molecular bases of conformation traits in dairy cattle.

## Figures and Tables

**Table 1:** Descriptive statistics for Final Classification (FC), Dairy Strength (DS), Rump, Feet and Legs (FL), and Mammary System (MS) in Brazilian Holstein cows.

Variable	N	Mean	±SD	Minimum	Maximum
FC	2339	101.35	3.21	89	111
DS	2339	100.93	2.59	88	108
Rump	2339	99.05	3.57	82	109
FL	2339	98.93	3.11	88	110
MS	2339	103.87	4.36	88	119

N—number of animals in the analyses; SD—standard deviation.

**Table 2:** Description of the significant genomic markers, candidate genes, and number of QTLs significantly associated with Final Classification (FC), Dairy Strength (DS), Rump, Feet and Legs (FL), and Mammary System (MS) traits in Brazilian Holstein cattle.

Trait	CHR	Position (bp)	−log_10_ (*p*-Value)	Genes (±100 kb)	QTLs
FC	10	58,643,603	8.02	*TMOD2*, *TMOD3*, *LYSMD2*, *SCG3*, *DMXL2*, ENSBTAG00000050593	-
	10	58,635,462	7.38	*TMOD2*, *TMOD3*, *LYMSD2*, *SCG3*, ENSBTAG00000050593	-
	26	44,637,034	8.22	*ZRANB1*, *CTBP2*	7
DS	3	63,243,334	7.55	*ADGRL2*	-
	5	112,981,457	7.75	*SREBF2*, *bta-mir-33a*, *SHISA8*, *TNFRSF13C*, *CENPM*, ENSBTAG00000050336, *SEPTIN3*, *WBP2NL*, *NAGA*, *PHETA2*, *SMDT1*, *NDUFA6*, *MGC127055*	47
	11	63,571,138	8.61	*RAB1A*, *ACTR2*	15
	12	31,640,020	8.54	*FLT1*	-
	19	5,726,386	7.54	*MMD*	1
	22	2,988,284	7.73	*ZCWPW2*	2
	25	35,085,041	7.46	*CUX1*, *MYL10*	7
Rump	5	47,431,158	8.62	*GRIP1*, *U1*, *HELB*, ENSBTAG00000053419	23
	5	105,666,159	7.72	*RAD51AP1*, *C5H12orf4*, *FGF6*, *FGF23*, *TIGAR*, *CCND2*	27
	10	93,552,272	7.60	ENSBTAG00000050021	-
	11	72,567,462	7.82	*TCF23*, *PRR30*, *ABHD1*, *PREB*, *CGREF1*, *KHK*, *EMILIN1*, *AGBL5*, *TMEM214*, *MAPRE3*, *SNORA62*	15
	11	63,571,138	7.64	*RAB1A*, *ACTR2*	
	19	5,726,386	7.77	*MMD*	1
	22	32,355,169	7.91	*FRMD4B*, *LMOD3*, *ARL6IP5*	1
FL	3	63,219,395	7.61	*ADGRL2*	-
	9	7,763,043	8.07	*ADGRB3*	3
	14	61,736,112	9.26	ENSBTAG00000052148, *KLF10*	27
	22	6,375,507	8.14	*OSBPL10*	15
	23	17,018,464	7.91	*ZNF318*, *ABCC10*, *DLK2*, *TJAP1*, *LRRC73*, *YIPF3*, *POLR1C*, *XPO5*, *POLH*	16
	23	44,473,942	7.41	*HIVEP1*	
MS	7	53,622,219	7.91	*FGF1*, *bta-mir-2460*, ENSBTAG00000042624	5
	11	78,083,413	7.60	*LDAH*	20
	12	85,218,262	7.54	*COL4A2*, *RAB20*, *NAXD*, *CARS2*, *ING1*	1
	28	14,058,016	9.36	*BICC1*	-

**Table 3:** Gene ontology terms related to the genes associated with Final Classification (FC), Rump, Feet and Legs (FL), and Mammary System (MS) in Brazilian Holstein cattle.

Trait	GO	Term	*p*-Value	Genes
FC	GO:0051694	Pointed-End Actin Filament Capping	0.002	*TMOD2*, *TMOD3*
	GO:0030239	Myofibril Assembly	0.003	*TMOD2*, *TMOD3*
	GO:0006936	Muscle Contraction	0.013	*TMOD2*, *TMOD3*
	GO:0007015	Actin Filament Organization	0.038	*TMOD2*, *TMOD3*
	GO:0005865	Striated Muscle Thin Filament	0.002	*TMOD2*, *TMOD3*
	GO:0030016	Myofibril	0.005	*TMOD2*, *TMOD3*
	GO:0005856	Cytoskeleton	0.074	*TMOD2*, *TMOD3*
	GO:0005523	Tropomyosin Binding	0.002	*TMOD2*, *TMOD3*
Rump	GO:0010628	Positive Regulation of Gene Expression	0.006	*EMILIN1*, *FGF23*, *FGF6*, *TCF23*
	GO:1905168	Positive Regulation of Double-Strand Break Repair via Homologous Recombination	0.020	*RAD51AP1*, *ACTR2*
	GO:0045737	Positive Regulation of Cyclin-Dependent Protein Serine/Threonine Kinase Activity	0.024	*CCND2*, *MAPRE3*
	GO:0008543	Fibroblast Growth Factor Receptor Signaling Pathway	0.050	*FGF23*, *FGF6*
	GO:0008284	Positive Regulation of Cell Proliferation	0.059	*CCND2*, *FGF23*, *FGF6*
	GO:0030154	Cell Differentiation	0.083	*FGF23*, *FGF6*, *TCF23*
	GO:0009887	Animal Organ Morphogenesis	0.092	*FGF23*, *FGF6*
	GO:0005737	Cytoplasm	0.027	*HELB*, *FRMD4B*, *TIGAR*, *ACTR2*, *C5H12orf4*, *CCND2*, *FGF23*, *FGF6*, *KHK*
	GO:0005856	Cytoskeleton	0.040	*ARL6IP5*, *FRMD4B*, *LMOD3*
	GO:0005881	Cytoplasmic Microtubule	0.049	*MAPRE3*, *TMEM214*
	GO:0035861	Site of Double-Strand Break	0.067	*HELB*, *ACTR2*
	GO:0005104	Fibroblast Growth Factor Receptor Binding	0.022	*FGF23*, *FGF6*
FL	GO:0016020	Membrane	0.021	*ABCC10*, *ADGRL2*, *OSBPL10*, *TJAP1*
	GO:0005829	Cytosol	0.076	*POLH*, *HIVEP1*, *XPO5*, *OSBPL10*, *ZNF318*
MS	GO:0031012	Extracellular Matrix	0.066	*COL4A2*, *FGF1*

## Data Availability

The data presented in this study are available on request from the corresponding author.
